# Entrustment of student supervision to GP trainees - a qualitative interview study of factors influencing entrustment decisions

**DOI:** 10.1186/s12909-025-07870-9

**Published:** 2025-09-12

**Authors:** Sabine Gehrke-Beck, Ylva Holzhausen

**Affiliations:** 1 Institute of General Practice, Universitätsmedizin Berlin, corporate member of Freie Universität Berlin and Humboldt-Universität zu Berlin, Charité Campus Mitte, Charitéplatz 1, 10117 Berlin, Germany; 2 Dieter Scheffner Center for Medical Education and Educational Research, Universitätsmedizin Berlin, corporate member of Freie Universität Berlin and Humboldt-Universität zu Berlin, Charité Campus Mitte, Charitéplatz 1, 10117 Berlin, Germany

**Keywords:** Medical education, Residency, General practice, Workplace-Based learning, Entrustable professional activities

## Abstract

**Background:**

The concept of Entrustable Professional Activities (EPAs) is being increasingly adopted for teaching tasks. However, it is debated how entrustment of teaching EPAs compares to entrustment of clinical EPAs. This study investigates the factors influencing the decision to entrust trainees with the supervision of medical students in general practice.

**Methods:**

Semi-structured interviews were conducted with nine general practitioners (GPs) and nine GP trainees. The interviews were transcribed and analyzed using the six steps of Braun and Clarke’s thematic analysis.

**Results:**

Entrustment of student supervision to GP trainees was influenced by factors related to the GP trainee, the GP, the relationship between the GP trainee and the GP, the teaching task, and the context. Trainee factors included enjoyment of teaching, familiarity with the practice environment, teaching skills and knowledge. Teaching skills were often considered as personality traits rather than learnable skills. Contextual factors included workload and availability of protected teaching time, and availability of students, teachers, and patients. Risks are seen in overburdening trainees and passing on incorrect knowledge.

**Conclusions:**

The entrustment of teaching EPAs is influenced by comparable factors as the entrustment of clinical EPAs. Didactic training could support trainees. Explicit entrustment and assessment by direct supervision are not established in the practice context but could help to minimize the risks associated with trainee teaching.

**Supplementary Information:**

The online version contains supplementary material available at 10.1186/s12909-025-07870-9.

## Background

Entrustable Professional Activities (EPAs) are advocated for training of clinical tasks and skills to improve safe and structured learning [1, 2]. Teaching in medical education also has been referred to as an “entrustable facet of professional work”, where physicians are entrusted with the qualification of learners [3]. The idea of the EPA concept is to define those authentic tasks of a profession that learners are expected to carry out without supervision at the end of a training phase and to structure the learning accordingly [2]. Defined entrustment-supervision levels reflect the skills and professional growth of trainees while minimizing risks of premature entrusting [4].

It has been proposed that the adoption of EPAs in teaching might enhance the quality of teaching in health professions [3, 5]. EPAs are especially useful, when risks are inherent [6]. Risks of failure in teaching in the clinical context include an unsafe learning environment for students and for patients [3] and passing on of incorrect knowledge. As wrong facts may be spread to future doctors, some argue that failure in teaching may even have wider consequences than incorrect clinical action [5]. Several examples of teaching EPAs have been published, covering either single teaching activities, or complete frameworks for teachers in undergraduate health professions or undergraduate medical education [3, 5, 7, 8]. According to the publication of Peters and colleagues [8], teachers are, for example, expected to perform 10 teaching EPAs related to classroom-based teaching (e.g. “give a lecture” or “teach a seminar”) and 3 teaching EPAs related to workplace-based teaching (e.g. “supervise a medical student in a short-block clinical placement”). Although these EPAs and frameworks have been developed and some have been implemented, the debate over whether the concept of entrustment actually applies to teaching is ongoing [9].

The aspect of entrustment is essential to the clinical EPA concept, as entrustment-supervision scales are applied to define, how autonomously a trainee can perform a specific task while ensuring patient safety. The widely applied entrustment-supervision levels range from being not allowed to perform EPA, to practicing the EPA under direct, indirect supervision or no supervision [10]. Numerous studies have been conducted to understand, which factors shape a supervising physician’s decision to let a trainee take care of a specific patient under a certain level of supervision. Skills and personality traits of trainees were discussed most prominently [11]. Other approaches aimed to develop more complex models of entrustment, in order to better understand how these factors might interplay in entrustment decisions [12, 13]. These works suggest that the decision to entrust trainees with a clinical EPA is influenced by factors related to (1) the trainee, (2) the supervising physician, (3) the relationship between the trainee and the supervising physician, (4) the EPA, and (5) the context.

Entrustment-supervision levels and entrustment decisions in particular could differ with respect to teaching EPAs. It is unclear, whether teachers are actually entrusted to perform the teaching EPAs, or whether they are assigned to teaching duties without prior assessment of their skills and independent of their qualifications. Iqbal and colleagues [14] propose that three entrustment supervision levels could be applied to evaluate, whether teachers have enough competence to perform teaching EPAs, while Bruggen and colleagues as well as Peters and colleagues excluded the discussion of supervision levels in their publications [7, 8]. Van Dam and colleagues developed an EPA for bedside teaching and suggest that novice teachers should be observed when teaching and get feedback, but it is unclear whether this approach is adapted in clinical settings [5].

The aim of the presented study is to gain a deeper understanding of the process of entrustment in the delegation of teaching responsibilities in a general practice setting. Trainees commonly supervise students in hospital and practice settings [15–17], and it has been defined as a potential EPA [7, 8]. This qualitative study analyzed the factors influencing the entrustment of general practice trainees (GP trainees) with the supervision of clerkship students, guided by existing entrustment models. Both the perspective of GP trainees and general practitioners (GPs) was obtained by means of guided interviews, as learners play an active role in entrustment decisions [18, 19].

## Methods

### Setting

The study recruited participants via a nationwide network of GP teaching (Gesellschaft für Hochschullehre in der Allgemeinmedizin). In Germany, medical undergraduate training includes a general practice clerkship in the clinical years. Some faculties offer additional clinical exposure in general practice in the preclinical years, and students can spend part of the final year clerkship in general practice. The recruitment and qualification of teaching practices is organized by the local faculty, and national position papers recommend mandatory didactic training [20, 21]. However, due to a lack of resources, didactic qualifications are not carried out regularly and comprehensively [21]. Further training to become a GP specialist prescribes periods of postgraduate training in various disciplines and in hospital as well as outpatient settings, but is not very structured beyond this. In most cases, trainees start their postgraduate training in hospitals and complete the sections in outpatient care later on.

### Study design

Semi-structured interviews were chosen over focus groups, as we assumed that experiences and reflections are reported more openly in a confidential setting. Between October and December 2023, semi-structured interviews were conducted with both GP trainees as well as GPs in general practices using a critical realistic approach [22, 23]. Study design and reporting was informed by the SRQR checklist [24], and the study protocol received approval from the Charité Ethics Board (No. EA_177_23 dated 30 August 2023). The data protection was reviewed by the data protection team of the Clinical Trial Office (CTO) of Charité – Universitätsmedizin Berlin.

### Participants

We used purposeful sampling to include GP trainees and GPs with different backgrounds and perspectives. We included GPs of varying age and gender as well as those with short vs. longer working and teaching experiences and trainees in their first years of residency as well as those with longer training and working experience. We recruited interviewees from different regions of Germany (Bavaria, Berlin, Thuringia, and North Rhine-Westphalia) and varying practice settings (single-handed practices vs. practices with several GPs). GPs were recruited via the nationwide network of University Teachers in General Practice (Gesellschaft für Hochschullehre in der Allgemeinmedizin GHA). We aimed to include pairs of GPs with their trainees, and GPs asked their trainees whether they were interested in participating. They were not informed whether their trainees did participate or not. We started transcribing and first coding while sampling the last interviewees and continued sampling until we had a satisfying representation of regions, age, and gender, as well as practice settings, and no new major aspects were expressed in two consecutive interviews.

### Data collection

Interviews were planned and suggested as video calls but were conducted on request in person or by telephone. The interviews were conducted by SG, a GP, lecturer, and teaching coordinator.

The interview guide consisted of open-ended questions and was informed by models of entrustment decisions from literature [13, 25, 26]. The interviews also covered aspects of the acceptability of near-peer teaching in practice settings, which were published in a separate article. The main questions of the interview guide are shown in Table 1; the complete interview guide can be found in the additional file 1. All interviewees received written study information and gave informed consent.

**Table 1:** Interview questions

Main questions for GPs	Main questions for GP trainees
What role does the trainee have in the daily practice routine as part of the team when students are in the practice?	What is your role in the daily practice routine as part of the team when students are in the practice?
What consequences/effects do you see when trainees supervise students (for students, the practice, patients, trainees, yourself)?	What consequences/effects do you see when you supervise students (for students, the practice, patients, yourself)?
How well are trainees prepared and able to supervise students?	How well prepared and able do you feel to supervise students?
What makes it difficult to involve trainees in student supervision?	What do you find difficult?
Is there anything else you would like to add? Have I forgotten to ask something?	Is there anything else you would like to add? Have I forgotten to ask something?

### Data analysis

Interviews were recorded using a digital voice recorder and transcribed verbatim and anonymized. Transcripts were analyzed using the six steps of thematic analysis according to Braun & Clarke [27, 28]. After data familiarizing with repeated reading and formulation of short case descriptions (step 1), first codes were created deductively from entrustment decision models (step 2). Subthemes were defined inductively from the material. First coding was done by SGB and started during the data collection to guide an informed decision for saturation. Themes and subthemes were developed from first coding in repeated meetings (YH, SGB) (step 3). Both authors independently coded a subset of interviews and resolved discrepancies. Thus, a final coding framework was refined consented. SGB reviewed the citations of each theme and subtheme (step 4). The revised themes and subthemes were finalized (Step 5), and exemplary citations were added (see Table 2).

**Table 2:** Coding framework with themes and subthemes

**GP Trainee factors**	
Joy of teaching	“If my new trainee doctor enjoys it, I'll support that too.” GP8
Teaching skills	“I think it's incredibly important that they first learn how to give constructive feedback as part of a training course. And that they then practice this with real students.” GP2 “We don't organize courses for trainees on how to teach - that's in our genes, so you don't have to explain it; it's automatic.” GP4
Familiarity with the practice environment	“And if they're new, then it is not possible; they have to get used to us here first, learn our processes, um, that is, once they've been here for six months, got their feet under them, got to know us all, got the computer program running, then we're very happy to do something like that. So, I don't think you can generalize like that; they have to settle in first.” GP1
Professional knowledge and expertise	“So, as far as qualifications are concerned, I think the training assistants we've supervised so far have all been very committed to their own training-so close to their own training that they're already good at it, I think.” GP7
**GP factors**	
Wish for support	“And for me it's, when you're in a single-handed practice and you have the students at your side all week and all the time, it's extremely exhausting, and it's nice when it's divided among us[trainee and GP].” GP2 “And we also force them to change a bit, so that they're not just with one doctor all the time. Um, I think they benefit from that.” GP1
Experience with near-peer teaching	“That´s how I have experienced it myself. I found it best, when someone is very close to my level of training, almost exactly the same, just a few steps ahead.” GP1
Relationship of GP trainee and GPs	“And the trainee can't be integrated into the care either, because you don't know her yet and can't assess her at all.” GP1
**Tasks characteristics**	
Students’ characteristics	“I find it a little easier with those who are in their final year or who come to us for a block placement. With the others [in first and second year], I think it's difficult for them to differentiate between what they can and can't be entrusted with.” GP6
Time-consuming learning activities	“Well, the Mini Mental or the DemTect, that's a longer test that I can't do alone with them, I can't manage to do it with them, and then I often say, yes, do it and then discuss it together [trainee and student] and then, or with me. They [trainees] often have more time for longer things like that.” GP1
**Context-factors**	
Workload	“If they are now, for example, with the rush, e.g., Monday in the consultation hour, when we realize that they are actually totally stressed, then we tend not to give them the students.” GP5
Availability of protected teaching time	“My absolute negative example is trainees in the clinic, who are forced into student teaching after 4 pm. If you want to implement this in the outpatient sector, which I think is good, you also have to structure it in such a way that time is set aside for it.” GP6 “Another aspect that I think is important is how much space I have for it. I think that's a very crucial point. If you want trainees to contribute there, then there has to be explicit space for it.” T6
Availability of students	“Especially since sometimes there are a lot of students. So, we've had two students here recently, and then it's inevitable that one of them will be with me.” T2
Availability of teachers	“…because, for example, in the afternoon I'm the only one, not the only one, but one of two, who is there in the afternoon, so that I've taken someone with me.” T5
Patients	“Because my colleague is more of a geriatric specialist, I'm more of an internist and sports medicine specialist, and that’s why they also rotate with the trainees.” GP6
**Risks for GP trainee and students**	
Overburdening the trainees	“And then I'm worried, let's say, if I have a student with a trainee for a whole week, I think I'd be worried that they'd be completely exhausted by the end of the week.” GP5
Students learning incorrect facts	“…and I have to keep an eye on that, and sometimes I worry that students are learning the wrong things.” GP5 “And of course, I don't want to pass on any false knowledge. So, you've made up your mind on how to explain some things or explain them to laypeople, and of course I don't want to say anything wrong.” T2.

*GP* General Practitioner, *T* Trainee

The quotations were translated with the help of DeepL Translate, and both authors fully checked and revised the translations. DeepL Translate was used to improve the English in the manuscript (step 6). All suggested wording and text passages were thoroughly reviewed and modified.

### Reflexivity

The interviews were conducted by the first author, who is a general practitioner, lecturer, and teaching practice coordinator. Analysis was supported by the last author, who is a medical education researcher with expertise in EPAs.

In order to bring a broader perspective into the survey and analysis, both the data collection and the data analysis were discussed in a research group, integrating both the medical didactic and educational research perspectives as well as the general medical practice and continuing education perspective. The study design and coding guidelines were discussed in the research meetings of the Institute of Educational Research and Didactics, and the interview guidelines and results were discussed in the research workshop of the Institute of General Practice with GPs, doctors in further training, health services researchers, and university lecturers. This ensured the traceability of all survey and evaluation steps, and appropriate adjustments were made iteratively.

## Results

Nine GP trainees and nine GPs participated in interviews. We could include seven pairs of trainees and their supervising GPs, one GP with no trainee volunteering to participate, and one GP with two trainees agreeing to be interviewed. The characteristics are displayed in Table 3. Each interview lasted between 45 and 66 min (mean: 58 min).

**Table 3:** Characteristics of interviewees

Characteristic	GPs (*n* = 9)	GP Trainees (*n* = 9)
Gender		
Female	4	5
Male	5	4
Age group		
20–29 yrs	0	1
30–39 yrs	1	7
40–49 yrs	1	1
50–59 yrs	4	0
≥ 60 yrs	2	0
Not available	1	0
Duration of GP training		
1–3 yrs	–	3
4–6 yrs	–	6
Teaching and training experience		
< 5 yrs	1	–
5–10 yrs	2	–
> 10 yrs	6	–
Size of GP practice		
Single-handed practice	1	1
Practice with 2–4 GPs	4	4
Practice with > 4 GPs	4	4
Location of practice		
City (> 100,000 inhab.)	6	6
Rural or small town	3	3

From the analysis of the transcribed interviews, various factors influencing the entrustment decisions were identified. Factors related to trainees, learning tasks, and GPs were predominantly raised by GPs; context factors and perceived risks involved were discussed by GPs as well as GP trainees. The complete coding framework can be found in Table 2.

GP Trainee factors.

Several factors relating to trainee characteristics were mentioned by the GPs as important for entrustment of student supervision, as, for example, the joy of teaching.

“if my new trainee doctor enjoys it, I’ll support that too.” GP8.

GP trainees’ teaching skills were also seen as important. They were often regarded as a personality factor rather than a competence to be acquired through training, although some mentioned didactic training as helpful.

“We don’t organize courses for trainees on how to teach - that’s in our genes, so you don’t have to explain it; it’s automatic.” GP4.

“I think it’s incredibly important that they first learn how to give constructive feedback as part of a training course. And that they then practice this with real students.” GP2.

Another prerequisite for supervising students was familiarity with the practice environment. Trainees needed to be confident and aware of the day-to-day procedures in the practice.

“…and if they’re new, then it is not possible; they have to get used to us here first, learn our processes, um, that is, once they’ve been here for six months, got their feet under them, got to know us all, got the computer program running, then we’re very happy to do something like that. So, I don’t think you can generalize like that; they have to settle in first.” GP1.

The GPs had little concern that the GP trainees did not have enough professional knowledge and expertise to supervise students. They presuppose that most trainees bring along some professional experience from preceding residency posts. If they start their residency in practice, they would not let them teach right away.

“So, as far as qualifications are concerned, I think the training assistants we’ve supervised so far have all been very committed to their own training, so close to their own training that they’re already good at it, I think.” GP7.

GP Factors.

GPs often expressed their wish for support by sharing supervision of students for time relief but also for varying learning opportunities for students.

“And for me it’s, when you’re in a single-handed practice and you have the students at your side all week and all the time, it’s extremely exhausting, and it’s nice when it’s divided among us [trainee and supervisor].” GP2.

“and we also force them to change a bit, so that they’re not just with one doctor all the time. Um, I think they benefit from that.” GP1.

One GP cites his own experience with near-peer teaching as positive.

“…and that´s how I have experienced it myself. I found it best, when someone is very close to my level of training, almost exactly the same, just a few steps ahead.” GP1.

Relationship of GP trainee and GPs.

Aspects of the relationship between GP trainees and GPs are seldom explicitly mentioned.

“And the trainee can’t be integrated into the care either, because you don’t know her yet and can’t assess her at all.” GP1.

Task characteristics.

Entrustment also depended to some extent on the characteristics of the student, whom the trainee should supervise. Some GPs felt more comfortable entrusting supervision of more advanced students as their level of knowledge and experience was closer to trainees.

“I find it a little easier with those who are in their final year or who come to us for a block placement. With the others [in first and second year], I think it’s difficult for them to differentiate between what they can and can’t be entrusted with.” GP6.

Time-consuming learning activities such as instructing and supervising preventive checkups or structured geriatric assessments were willingly transferred to trainees as they were considered to have more time available for teaching.

“Well, the Mini Mental or the DemTect, that’s a longer test that I can’t do alone with them, I can’t manage to do it with them, and then I often say, yes, do it and then discuss it together [trainee and student] and then, or with me [trainees] often have more time for longer things like that.” GP1.

Context factors.

In the GP practice setting, workload and availability of protected teaching time are cited as factors that influence entrustment. If there is a particularly high workload on the side of the trainee, teaching is not delegated to them.

“If they are now, for example, with the rush, e.g., Monday in the consultation hour, when we realize that they are actually totally stressed, then we tend not to give them the students.” GP5.

Conversely, attempts are made to ensure the availability of protected teaching time for trainees so that they can provide adequate student supervision.

“My absolute negative example is trainees in the clinic, who are forced into student teaching after 4 pm. If you want to implement this in the outpatient sector, which I think is good, you also have to structure it in such a way that time is set aside for it.” GP6.

GP trainees also support the GPs’ proposition that protected teaching time needs to be available.

“Another aspect that I think is important is how much space I have for it. I think that’s a very crucial point. If you want trainees to contribute there, then there has to be explicit space for it.” T6.

Trainees also describe the availability of students as a factor as well as the lack of availability of teachers.

“Especially since sometimes there are a lot of students. So, we’ve had two students here recently, and then it’s inevitable that one of them will be with me.” T2.

“…because, for example, in the afternoon I’m the only one, not the only one, but one of two, who is there in the afternoon, so that I’ve taken someone with me.” T5.

Some GPs had additional qualifications and were consulted by a specific subgroup of patients. They considered the patients that presented in the trainee’s consultation hours as more appropriate for students, as they presented with a wider selection of complaints and diseases.

“Because my colleague is more of a geriatric specialist, I’m more of an internist and sports medicine specialist, and that’s why they also rotate with the trainees.” GP6.

Risks for GP trainees and students.

Only some of the interviewees are worried when using trainees in student teaching. The GPs see two main risks: overburdening the trainees and students learning incorrect facts - with the first risk being mentioned more frequently.

“And then I’m worried, let’s say, if I have a student with a trainee for a whole week, I think I’d be worried that they’d be completely exhausted by the end of the week.” GP5.

“…and I have to keep an eye on that, and sometimes I worry that students are learning the wrong things.” GP5.

The GP trainees also fear that students might learn incorrect facts.

“And of course, I don’t want to pass on any false knowledge. So, you’ve made up your mind on how to explain some things or explain them to laypeople, and of course I don’t want to say anything wrong.” T2.

## Discussion

This study identified several factors that contributed to GPs’ decisions to entrust student supervision to GP trainees: factors related to the GP trainee, the GP, the GP-trainee relationship, the task, the context, and the perceived risks involved.

When compared to the model of Gin and colleagues [12], categories of factors involved in teaching entrustment are comparable to those seen in clinical entrustment, while the individual factors show some differences. The model defines, for example, specific trainee factors-agency, reliability, integrity, capability and humility-to influence entrustment decisions [12]. In this study, GPs reported that the trainees’ enjoyment of teaching, as well as their teaching skills, familiarity with the practice environment, and knowledge and experience, influenced the decision to entrust them with student supervision. Most of these factors could be summarized as the trainee’s capability, whereas the trainee’s enjoyment of teaching could be related to the trainee’s agency. Reliability, integrity, and humility, which play an important role in clinical entrustment, were not explicitly mentioned [29]. In clinical entrustment models, GP factors are subdivided into trust propensity, experience, accountability, ability to support, and benevolence [12]. In teaching entrustment analyzed here, GPs influenced the decision with their desire for support in teaching and their own experience of teaching near peers. The desire for support may be related to the GP’s propensity to trust, but the GP’s accountability, ability to support, or benevolence were not explicitly mentioned. The relationship between the GP and the trainee was acknowledged as important, but no specific relationship factors were detailed.

Among the factors related to task characteristics, student maturity was considered. Students in later clinical years were considered less difficult to supervise because their skills were more advanced, and therefore they could be more easily integrated into patient care. This student-related factor appears to be similar to the effect of patient presentation on entrustment in clinical EPAs. The finding that the supervision of more time-consuming learning activities was more often delegated is a factor that has not previously been discussed in clinical entrustment, but time expenditure required for patient care may also be relevant to the entrustment of clinical tasks.

With respect to context factors, the workload and the availability of teachers are factors influencing the entrustment decision, which are also known with respect to clinical EPAs [12]. The availability of protected teaching time for trainees and the unavailability of the GP for student supervision were frequently mentioned in interviews with both GP trainees and GPs.

Both GPs and GP trainees mentioned the perceived risks associated with entrusting GP trainees with the supervision of students. These perceived risks are also relevant when entrusting trainees with clinical EPAs. Direct risks to patients seemed negligible, but the risks of passing on incorrect knowledge with possible indirect risks to patients were mentioned. Lack of knowledge on the part of trainees was not a major concern for most respondents. It was important to them that trainees felt confident working in the practice environment. Specific knowledge gaps were not seen as a barrier to teaching, as trainees could always ask their supervisors questions. The risk of overburdening trainees was discussed more frequently. Resident burnout is a concern in Germany and other countries [30] and can be associated with medical errors and therefore impact patient safety [31]. This risk appears to be addressed by making teaching assignments dependent on the workload and time available for the trainee to teach. In addition, enjoyment of teaching was an important factor in entrusting students with teaching, which may also reduce the risk of trainees perceiving teaching as too demanding. Teaching has been found to be challenging with competing clinical tasks [32, 33], and self-selection of those with positive attitudes toward teaching may alleviate the burden [34]. On the other hand, teaching is associated with many perceived benefits for trainees and junior doctors [35], and self-selection may prevent those less confident or without prior experience from benefiting from teaching involvement.

**Fig. 1:**
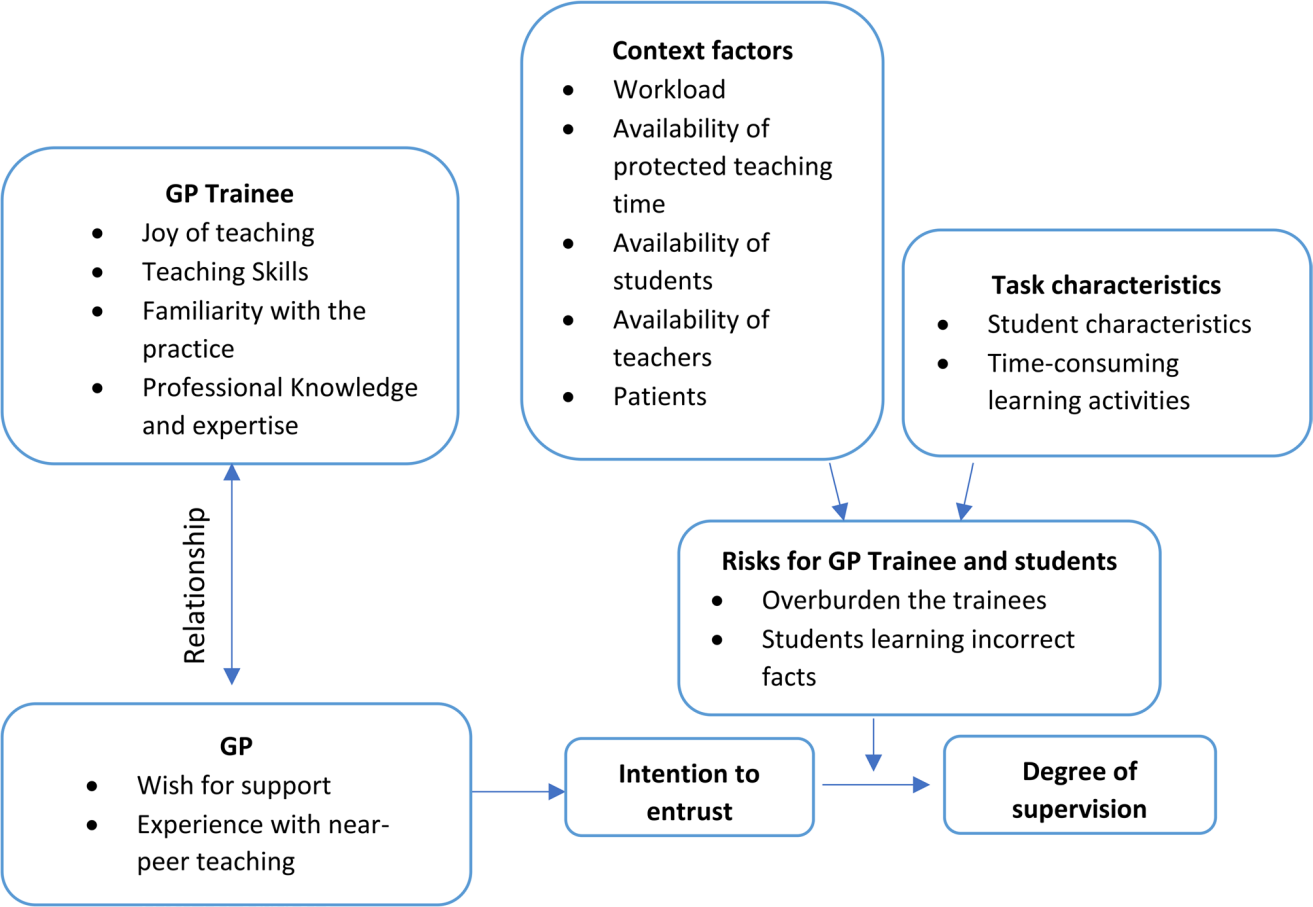
Schematic model of the factors influencing the GPs decision to entrust GP trainees with the supervision of students

The results of the present study show that the concept of entrustment applies to the delegation of teaching responsibilities in general practice. Family physicians in this study weigh several factors in deciding whether a particular trainee can be entrusted with student supervision. Figure 1 integrates the factors found in a schematic entrustment model, which is based on the clinical entrustment models [12, 13].

Notably, the GP trainees in this study who were entrusted with the supervision of students were themselves working under indirect supervision. None of the respondents described direct observation of the supervision of GP trainees to inform the entrustment decision, as suggested by Iqbal [36]. The decisions described often appear to be implicit, resulting from the self-selection of trainees with a love of teaching and a contextual need for teaching support. Observation of student supervision would indicate whether trainees have sufficient knowledge and didactic skills, as well as assess strain and overload. In a busy general practice context, direct observation may seem time-consuming and not easily feasible, but it could help to make a more informed decision and minimize the risks of trainees teaching.

In the interviews, enjoyment of teaching and teaching competence were often seen as personality traits and talents. Teaching competence is often misunderstood as simply “loving to teach” [3], neglecting the need for necessary qualifications and the possibility to support those who want to be better prepared for teaching. In order to minimize the risks of teaching, didactic input on how to deal with uncertainty can be helpful. Didactic guidance should be offered to those who are more hesitant and may allow more trainees the opportunity to teach. Trainees often report that they did not receive training for teaching [17, 33], and those who are offered training feel more competent and teach more often [35, 37].

Based on these results, postgraduate training programs in general practice should explicitly include teaching as a skill to be learned and practiced. Teacher training programs should include concepts for entrusting student supervision as a defined teaching activity. The courses could address risks and promote direct supervision. Due to time constraints, recording teaching sessions and providing subsequent feedback could improve implementation. Teaching EPAs could be included in medical school prior to postgraduate training. Since many medical schools offer peer teaching programs, teaching EPAs could be incorporated into these programs so that junior doctors are familiar with the concept before starting their postgraduate training.

This study adds to the knowledge of teaching entrustment decisions at the micro level of individual GP practices and allows the perspective of both GPs and GP trainees to be assessed. A selection bias towards practices that are more involved in teaching and training cannot be ruled out, as the topic of the interview was known and participation was voluntary without incentives. With respect to the factors identified, it cannot be ruled out that other factors are influencing GPs decision to entrust GP trainees with student supervision, such as the trainees’ reliability and humility. Further research is needed to complement the findings of this study. The findings are also limited to teaching tasks related to the supervision of students in the general practice setting. Further research is needed to determine whether these findings are generalizable to other teaching EPAs and settings.

## Conclusions

The decision to entrust trainees with student supervision was reported to be influenced by the same categories of factors that are also relevant in the entrustment of clinical EPAs, with the underlying factors showing some variation. GP trainees’ enjoyment of teaching and contextual factors such as patient volume and the availability of GPs and trainees with time resources for teaching were mentioned most often. Delegation of teaching often seemed implicit. Better awareness, reflection, and communication of the entrustment could promote a more explicit decision. Assessment of the trainee’s capability and confidence by direct observation could guide an informed and explicit decision and minimize the associated risks for students, patients, and trainees, although time resources for this approach may be a problem for feasibility. It may be helpful to consider didactic skills as a competence to be trained for safer teaching, rather than an innate personality trait and promote opportunities for qualification. Training and support for trainees and GPs could improve the teaching experience and allow more trainees to teach, with the benefits that this brings.

## Supplementary Information

Supplementary Material 1.

## Data Availability

The datasets generated and analysed during the current study are not publicly available as the participants of this study did not give written consent for the audiotapes and the transcripts of the interviews to be shared publicly but are available from the corresponding author on reasonable request.

## Abbreviations

EPA: Entrustable professional activity
GP: General practitioner
