# Supplementary material for: Entrustment of student supervision to GP trainees - a qualitative interview study of factors influencing entrustment decisions
Source: BMC Med Educ. 2025 Sep 12;25:1253. doi: 10.1186/s12909-025-07870-9 (PMC12427109; doi:10.1186/s12909-025-07870-9)
Supplement: Supplementary file 1 — Supplementary Material 1. [file 12909_2025_7870_MOESM1_ESM.docx]

| **Information**  **Concept of the theoretical framework** | **Questions for GP trainees** | **Questions for GPs** |
| --- | --- | --- |
| Sociodemographic information  Professional experience  Practice information  Practice type, services, practice customers | Short questionnaire | Short questionnaire |
| Practice routine - consultations  Practice routine - teaching and postgraduate training  Entrustment decision:  Learner  Teacher  Context  Relationship | What is your role in the daily practice routine as part of the team?  What is it like when students are in the practice?  to be clarified/ask if necessary:  *How often or regularly?*  *On whose initiative? (How did this come about?)*  *What experiences have you had/collected in other postgraduate education phases previously?*  *Have you supervised/taught students in postgraduate education to date?*  *Follow-on questions: in an inpatient setting? Other practices? Experienced differently?* | What role does the trainee have in the daily practice routine as part of the team? What is it like when students are in the practice?  to be clarified/ask if necessary:  *what involvement in supervision? What tasks? How often or regularly?*  *On whose initiative was the involvement? (How did this come about?)*  *Have you previously involved other trainees in student supervision?*  *Follow-on questions: How regularly? Depending on what? (Trainee/duration of postgraduate training/practice factors?)* |
| Advantages/ positive outcomes  Entrustment Decision:  Teacher, Learner Context, Complexity of task | What consequences/effects do you see when you supervise students (advantages or disadvantages)?  Follow-on questions:  *For students?*  *For the practice/the GPs?*  *For patients?*  *For yourself?* | Why do you involve trainees in student supervision? / What advantages/effects do you see when trainees supervise students?  Follow-on questions:  *For students?*  *For trainees?*  *For patients/practice?*  *For yourself?* |
| Competence  Entrustment Decision:  Learner | How well prepared and able do you feel to supervise students?  Follow-on questions: *prepared by what? Support? Needs?*  *Previous teaching experience?* | How well are trainees prepared/able to supervise students?  Follow-on questions:  *Depending on what? Previous experience, duration of postgraduate education, and personality? Area of activity?* |
| Barriers  Entrustment Decision:  Teacher, Learner Context, Complexity of task | What do you find difficult?  If necessary, ask:  *Confidence of the GP?*  *Your own confidence?*  *Rejection by students?*  *Lack of time/patient care more important?*  *Effort to familiarize yourself/find out information?*  *Feel comfortable with the task/fun?*  *Disadvantages for patient care?*  *For your own postgraduate education?*  *For the training of students?* | What makes it difficult to involve trainees in student supervision?  If necessary, ask:  *Confidence of trainees?*  *Your own confidence that trainees can take this on? Rejection by students?*  *Lack of time/patient care more important?*  *Effort to instruct trainees on this?*  *Disadvantages for patient care?*  *For further training for trainees?*  *For training students?* |
| Conclusion | Is there anything else you would like to add? Have I forgotten to ask something? | Is there anything else you would like to add? Have I forgotten to ask something? |

|  |  |  |
| --- | --- | --- |

Supplement 1: Interview Guide (Questions related to entrustment decision only)
